# Human-Validated Neural Networks for Precise Amastigote Categorization and Quantification to Accelerate Drug Discovery in Leishmaniasis

**DOI:** 10.1021/acsomega.4c08735

**Published:** 2024-12-24

**Authors:** Andrey Gaspar Sorrilha-Rodrigues, João Lucas Aparecido Rocha Paes, Yasmin Silva Rizk, Fernanda da Silva, Rafael Francisco Rosalem, Carla Cardozo Pinto de Arruda, Carlos Alexandre Carollo

**Affiliations:** † Laboratory of Natural Products and Mass Spectrometry (LAPNEM), Faculty of Pharmaceutical Sciences, Food, and Nutrition (FACFAN), Federal University of Mato Grosso do Sul, Campo Grande, Mato Grosso do Sul 79070-900, Brazil; ‡ Geomatics Laboratory - georeferencing and computer vision, Faculty of Computing, Federal University of Mato Grosso do Sul, Campo Grande, Mato Grosso do Sul 79070-900, Brazil; § Human Parasitology Laboratory, Institute of Biosciences, Federal University of Mato Grosso do Sul, Campo Grande, Mato Grosso do Sul 79070-900, Brazil

## Abstract

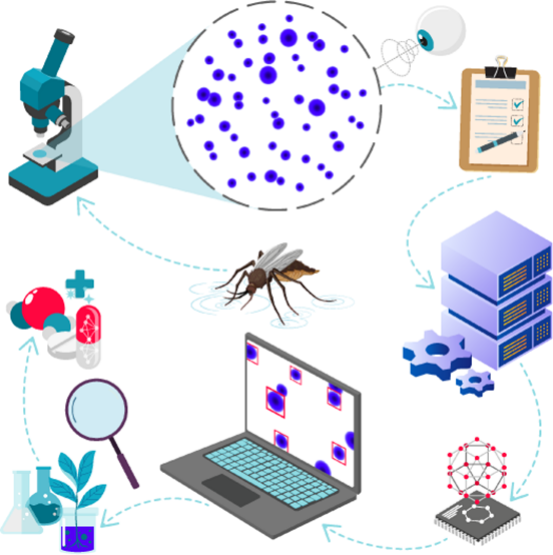

Leishmaniases present a significant global health challenge with limited and often inadequate treatment options available. Traditional microscopic methods for detecting Leishmania amastigotes are time-consuming and error-prone, highlighting the need for automated approaches. This study aimed to implement and validate the YOLOv8 deep learning model for real-time detection, quantification, and categorization of Leishmania amastigotes to enhance drug screening assays. YOLOv8 was trained on 470 images from two microscopes, classifying them into categories such as “infected cells,” “intracellular amastigotes,” “uninfected cells,” and “edge cells.” The model’s performance was compared to human operators using Pearson and Spearman correlation analyses. YOLOv8 achieved strong performance in detecting “infected cells” (AUC = 0.934) and “intracellular amastigotes” (AUC = 0.846). However, challenges remained in differentiating extracellular amastigotes from background noise (AUC = 0.672). Despite these challenges, the YOLOv8 model effectively minimized human variability in drug screening, providing a reliable and efficient tool for the quantification and categorization of Leishmania amastigotes in drug discovery efforts. While further refinements are required to resolve misclassification issues, the model demonstrates significant potential in enhancing both accuracy and throughput in preclinical assays.

## Introduction

Leishmaniases, as defined by the World Health Organization (WHO), make up a group of neglected tropical parasitic diseases (NTDs) caused by Leishmania protozoan parasites. They remain a global public health challenge because of their prevalence in tropical and subtropical regions, particularly in the most impoverished areas of the world. Such distribution requires a careful analysis of its various clinical manifestations and the obstacles encountered in treatment. The diversity of clinical forms of leishmaniasis, combined with the increasing resistance to existing medications, highlights the need to explore new therapeutic options.^1^

The current treatment for these diseases is based on chemotherapy as there are no vaccines available for humans. Pentavalent antimonials and liposomal amphotericin B have been the most widely used compounds in clinical practice, as well as other options such as miltefosine, paromomycin, and pentamidine.^2^ However, limitations remain in terms of cost, toxicity, and reduced efficacy of drugs over time.^2,3^

Primary screening often targets the promastigote form due to the ease of culture and handling, but this is not the target form found in the vertebrate host.^4^ A model for cultivating axenic amastigotes has been developed, but there are limitations as it does not cover many aspects of the parasite’s intracellular development.^5^ The tested compounds must be able to cross host cell membranes and reach the intracellular amastigotes, maintaining their stability at low pH. In addition, some compounds may require metabolism by the macrophage to exhibit activity or the macrophage itself can be directly targeted to induce intracellular parasite killing. Despite the limitations, the test against intracellular amastigote forms has been the most reliable method for evaluating the antileishmanial activity of compounds of natural, synthetic, or nanotechnological origin.

While microscopic examination remains a widely used method for this testing, it places a substantial burden on laboratory technologists, often resulting in errors and delays due to the labor-intensive and repetitive nature of the process.^6^ Typically, counting amastigotes within host cells requires the enumeration of amastigotes across 200 cells per slide to derive an average parasite count per host cell. The half-maximal inhibitory concentration (IC_50_) is a key metric for evaluating the efficacy of treatments against leishmaniasis, providing a quantitative measure of a compound’s potency. It helps select new prototypes and optimize therapeutic regimens by allowing precise comparisons between antileishmanial agents.^7^

However, accurately determining IC_50_ values presents challenges due to variability in experimental conditions, cellular context, and potential assay biases. Analyst variability and reproducibility issues further complicate the process as the experience and technique of the analyst can significantly impact the reliability of measurements. The manual counting of hundreds of cells across numerous slides increases the risk of errors and slows down the evaluation of compounds, making the process time-consuming and inconsistent, significantly limiting the ability to efficiently screen large numbers of compounds.^8^

In recent studies, models like LeishFuNet^9^ and DeepLeish^10^ have employed deep learning approaches to detect Leishmania amastigotes in microscopic images. LeishFuNet, using transfer learning with pretrained models, focused primarily on patient-level diagnostics rather than slide-level detection or quantification. Although single-class detection models typically outperform multiclass models,^11^ DeepLeish, which applied YOLOv5 for amastigote detection, achieved a mean average precision of 73%, highlighting the significant challenges inherent in this type of analysis. Despite these advances, neither model is suitable for drug screening, as they lack the ability to accurately quantify or categorize amastigotes, both crucial for evaluating the efficacy of new compounds.

Recognizing these bottlenecks underscores the need for automated parasite counting and enhanced evaluation methods. Advances in artificial intelligence (AI), particularly object categorization, provide promising solutions. In this study, we implemented YOLOv8, for real-time detection, categorization, and counting of intracellular Leishmania amastigotes. A key advancement in our approach is the integration of human validation alongside the automated model, ensuring that the AI-generated results align with expert evaluations. This human validation strengthens the robustness of the system, addressing concerns about model overestimation or underestimation of parasite counts. Our work builds upon previous research but significantly extends the application of AI to drug discovery by incorporating multiclass detection and real-time feedback, enabling more efficient, accurate, and validated evaluation of treatment efficacy compared to models such as LeishFuNet and DeepLeish.

## Results and Discussion

The “infected cell” and “intracellular amastigote” categories are essential tools for quantifying parasite load and assessing the antileishmanial activity of new drugs through the calculation of the IC_50_. These two categories are common tools for indicating antileishmanial activity. In the research of new treatments against Leishmania, accurate counting of these two categories allows scientists to quantify the impact of a drug on the reduction of the parasitic load in host cells. The “infected cell” category shows the presence of the parasite within the host cells, indicating the extent of infection, and the “intracellular amastigote” category represents the parasitic load itself. The accuracy in identifying these categories directly impacts the evaluation of a treatment’s efficacy and decisions about the progression of new drug candidates. Meanwhile, the categories “uninfected cells,” “extracellular amastigotes,” and “edge cells” serve specific functions within the classification model, helping avoid confusion with the main categories – “infected cells” and “intracellular amastigotes.” The “background” category, on the other hand, represents all elements of the image that do not belong to these defined classes. Although not directly labeled or annotated during training, the model must learn to recognize “background” regions indirectly, as these areas are not the primary focus of detection but are crucial for distinguishing relevant cellular structures.

Uninfected cells are those that show no presence of Leishmania amastigotes and are an important indicator of the parasite’s nonproliferation under treatment. They are significant because they provide a counterpoint to infected cells, allowing for the evaluation of a drug’s efficacy not just by the reduction of infected cells but also by the preservation of healthy cells. Therefore, these cells are considered in the calculation of IC_50_, and their correct identification is necessary to ensure accuracy in estimating the drug’s efficacy, as they indicate the proportion of cells that have not been affected by the infection or have a zero count of intracellular amastigotes.

While extracellular amastigotes indicate the presence of parasites, they are not factored into IC_50_ determinations, which specifically focus on the parasites within infected host cells. This is due to the nature of the assay, which targets intracellular amastigotes as the key measure for assessing the drug’s efficacy against Leishmania infection. This ensures that the evaluation is accurate and relevant to the parasite’s life stage in the host. The IC_50_ in this context measures the concentration of the drug needed to inhibit the biological effect, which in this study is the presence or absence of the parasite (parasitic load) and not the viability or reproduction of the parasite. Correctly identifying “extracellular amastigotes” helps avoid confusion with “intracellular amastigotes.” Since this research model focuses exclusively on intracellular amastigotes, counting extracellular amastigotes can lead to inaccurate results. If extracellular amastigotes are mistakenly counted as intracellular, it may lead to misinterpretations of the drug’s efficacy, potentially resulting in incorrect conclusions regarding its ability to control the infection.

Edge cells, when captured at the periphery of images, appear partially, which can result in incomplete data about the cell and any amastigotes that it might contain. Identifying edge cells is crucial for maintaining the integrity of the collected data. Partially visible cells can lead to interpretation errors such as overestimating or underestimating the number of infected cells and, consequently, the amastigotes present. This could negatively affect the accuracy of determining the IC_50_, as this metric depends on an accurate count of the reduction in parasites under different drug concentrations. Ensuring that the model properly identifies and classifies “edge cells” helps eliminate potentially inaccurate or misleading data that may arise from partially visible cells.

The YOLOv8 neural network allows for the real-time capture and analysis of microscopic images, providing the operator with the ability to conduct continuous and dynamic evaluation. This model not only records the discriminatory data from the images but also generates quantitative and qualitative results visually and instantly. As illustrated in Figure S1, a microscope equipped with a low-cost HY-500m camera was used, allowing the analyst to monitor the model’s performance during microscopic image capture.

**Figure 1:**
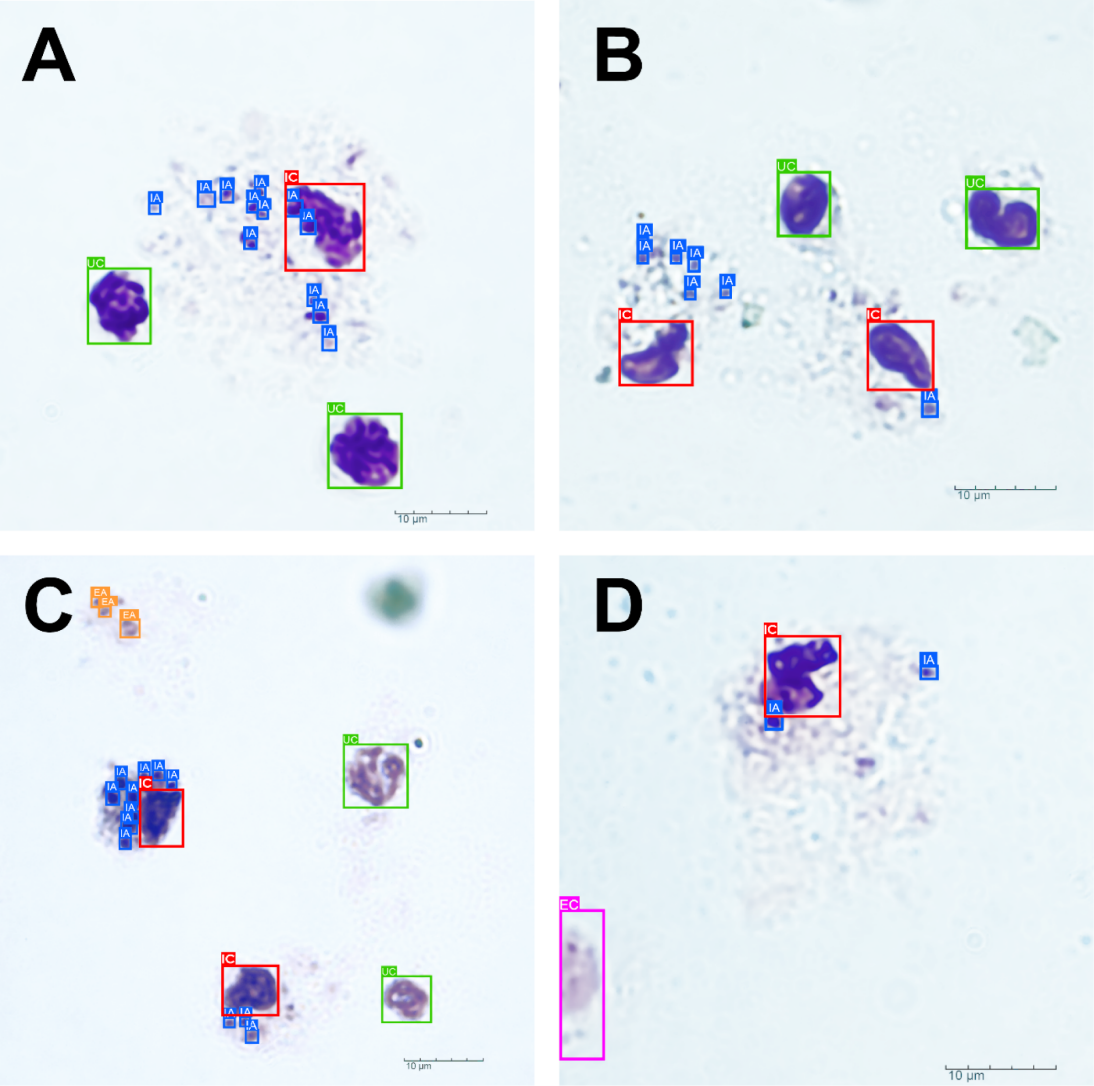
Examples of detection performed by the YOLOv8 model in microscopic images of cells associated with Leishmania infection. (A) “Infected cells” (IC—in red) and “intracellular amastigotes” (IA—in blue). (B) “Uninfected cells” (UC—in green). (C) “Extracellular amastigotes” (EA in orange). (D) “Edge cells” (EC—in pink).

Figure 1 presents a visual representation of the model’s effectiveness in detecting different states of cells associated with Leishmania infection. Figure 1A predominantly identifies cells as “infected cells” and “intracellular amastigotes,” detecting signs of infection and the presence of the parasite within the cells. Figure 1B focuses on “uninfected cells,” demonstrating the model’s ability to differentiate between infected and uninfected cells. Figure 1C features cells categorized as “extracellular amastigotes,” indicating the presence of amastigotes outside a host cell. Finally, Figure 1D illustrates “edge cells,” located at the periphery of the images, a common challenge for many detection models due to their partial position within the field of view.

Real-time detection can assist in the validation and continuous improvement of detection models by allowing developers and operators to directly identify where the model succeeds and where it fails, enabling specific adjustments that enhance its applicability and accuracy.^12^ This ensures that the model can be effectively used in laboratory contexts for evaluating Leishmania infection.

This immediate visualization streamlines work processes and promotes technology acceptance, complementing its implementation and facilitating the transition to automated practices. The ability to observe directly, combined with data comparison, reinforces healthcare professional’s confidence in the technology. Additionally, the system can serve as a training tool for new technicians and specialists given its accuracy and consistency in detection, establishing a benchmark for result interpretation.

**Figure 2:**
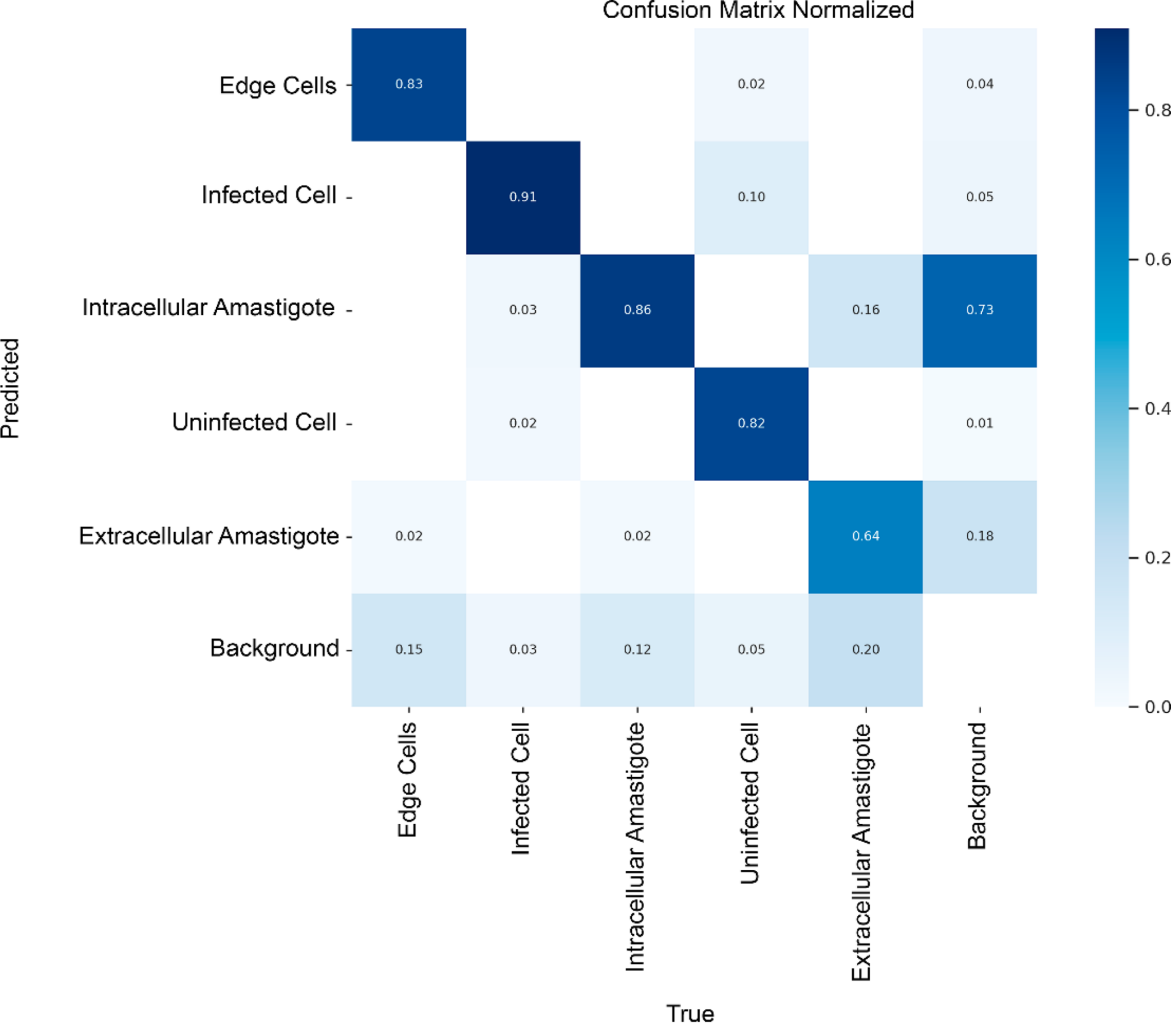
Normalized confusion matrix illustrating the model’s performance in classifying different cell and amastigote categories in microscopic images. The main diagonal indicates the percentage of correct predictions for each class, while the off-diagonal cells represent the confusion between categories. The categories include “edge cells,” “infected cells,” “intracellular amastigotes,” “uninfected cells,” “extracellular amastigotes,” and “background.” The color scale, ranging from white (0.0) to dark blue (1), serves as a visual indicator of the frequency of each prediction made by the model.

The normalized confusion matrix (Figure 2) provides a detailed view of the model’s classification rates for various conditions related to Leishmania in cells. The actual categories of the examples are organized in the columns, while the model’s predictions are arranged in rows. Each cell in the matrix reflects the proportion of how accurately or inaccurately the model classifies a given category. For instance, the cell at the intersection of the intracellular amastigote’ row and its corresponding column indicates that 86% of the true instances of this category were correctly identified. Lighter-colored cells indicate a lower frequency of correct or incorrect predictions, as exemplified by the 2% confusion between “intracellular amastigotes” and “extracellular mastigotes.”

The confusion matrix provides insight into the model’s performance by displaying metrics such as true positives (TP), true negatives (TN), false positives (FP), and false negatives (FN), allowing us to observe the frequency of each prediction. Despite the model’s high accuracy in detecting “infected cells” and “intracellular amastigote” categories, notable confusions arose with the image background and other cellular classes, similar to challenges reported by Gonçalves et al.^13^ This misclassification issue underscores the universal difficulties in microscopic image analysis, particularly in distinguishing between different cellular structures amidst background noise. Addressing these inconsistencies is crucial for iterative improvements to the model, ensuring better alignment between automated classifications and human evaluations.

For example, while the “infected cell” category achieved an accuracy of 91%, there was moderate confusion with “edge cells” (10%) and “background” (5%), highlighting areas for improvement in reducing false positives. This aligns with findings by Gonçalves et al.,^13^ where similar misclassifications occurred due to indistinct cellular structures. Refining the model to minimize these errors is vital for enhancing its utility in drug discovery, particularly in optimizing the evaluation of antileishmanial compounds.

The “intracellular amastigote” category achieved a correct classification rate of 86%, with the main classification errors occurring in the “extracellular amastigote” (16%) and “background” (15%) categories. These results indicate that while most amastigotes are correctly identified, there is some confusion with background elements, possibly due to low contrast in specific areas of the microscopic images.

The “edge cells” category was correctly identified 83% of the time, with the main confusions involving “background” (4%) and “infected cells” (2%). These results demonstrate good accuracy in detecting this category despite some residual confusion with other cellular structures.

The primary challenge observed with the “extracellular Amastigote” category lies in its frequent confusion with the “background” category, which accounted for 18% of misclassifications. This highlights a persistent issue in distinguishing amastigotes outside host cells from irrelevant background areas, which are not explicitly labeled during training but are integral to accurate detection. The presence of these false positives in the background significantly impacts the model’s reliability when differentiating between amastigotes and nonrelevant image regions.

Additionally, while the confusion with “intracellular amastigotes” was minimal at 2%, it underscores the importance of ensuring that extracellular amastigotes are not mistakenly counted alongside intracellular forms, as only the latter contribute to IC_50_ calculations. Addressing these issues through further model refinement and enhanced training is crucial for improving the detection accuracy and ensuring robust data for antileishmanial drug screening.

**Figure 3:**
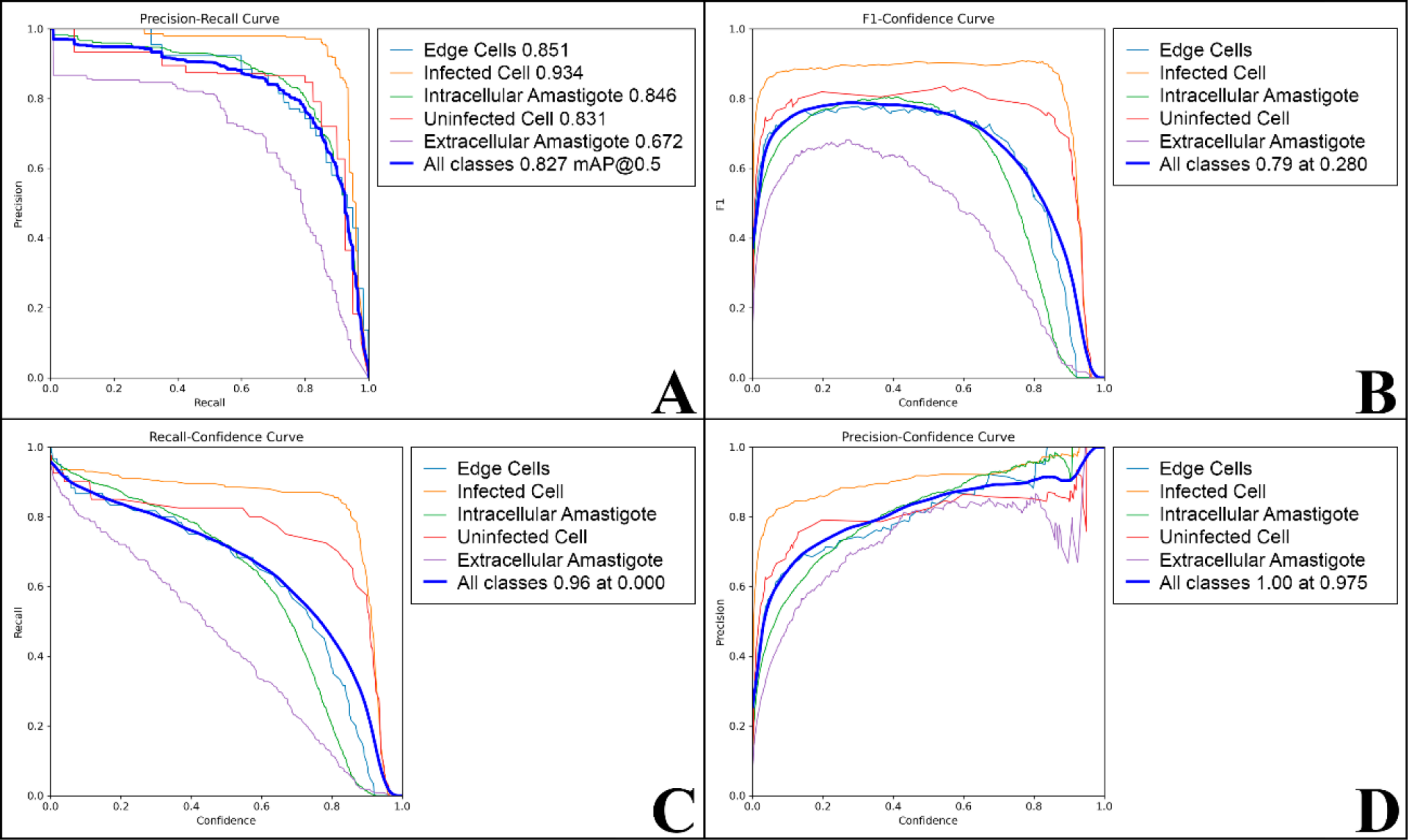
Performance graphs of the YOLOv8 model across multiple categories: “edge cells,” “infected cells,” “intracellular amastigotes,” “uninfected cells,” and “extracellular Amastigotes.” (A) Precision-recall, (B) F1-confidence, (C) recall-confidence, and (D) precision-confidence curves. These graphs demonstrate the relationship between the model’s performance metrics and confidence levels for each identified class.

The performance of the YOLOv8 model’s performance across multiple categories is illustrated through various evaluation curves, including precision-recall, F1-confidence, recall-confidence, and precision-confidence (Figure 3). These curves provide an in-depth analysis of the model’s behavior, offering insights into its accuracy across different metrics and confidence levels.

The precision-recall curve (Figure 3A) provides a clear view of the model’s performance across different classes. Notably, the “infected cell” class achieved the highest area under the curve (AUC) value of 0.934, indicating the model’s strong capability to accurately identify infected cells. This high precision is crucial as the accurate detection of infected cells is essential for evaluating the antileishmanial efficacy of new drug candidates. Meanwhile, the “intracellular amastigote” class showed an AUC of 0.846, reflecting good, albeit slightly lower, performance. Despite this, the ability to detect intracellular amastigotes remains vital for assessing the parasitic load within host cells, an important metric for determining the therapeutic response and overall drug efficacy against Leishmania. The precision in counting amastigotes directly impacts the reliability of treatment evaluations, underscoring the significance of these two categories in the drug discovery processes.

Other classes also performed well. The “edge cell” class recorded an AUC of 0.851, indicating that the model is effective in identifying cellular edges, which is essential for delimiting areas of interest in the images. The “uninfected cell” class obtained an AUC of 0.831, demonstrating the model’s ability to distinguish uninfected cells from other classes.

On the other hand, the lower AUC value of 0.672 for the “extracellular amastigotes” class suggests that the model may face challenges in accurately identifying this category compared to others. One possible reason for this performance could be confusion with the’background’ category, which includes any image regions not explicitly labeled as part of the main cell classes. Since extracellular amastigotes are small, free parasites outside the host cells, they may blend into or be misclassified as background noise due to their similar visual features. This overlap can reduce the model’s ability to differentiate extracellular amastigotes from irrelevant image areas, leading to lower precision and recall. A similar issue was highlighted by Gonçalves et al.^13^ and Sun et al.,^14^ where researchers faced challenges in distinguishing between the primary categories and background noise during model training for image classification tasks. Both studies underscore how background regions, which are not explicitly labeled, can lead to misclassification, particularly when the model struggles to differentiate small objects or detailed structures from irrelevant parts of the image. This problem is recurrent in histopathological image analysis and microscopic detection tasks, emphasizing the need for refined preprocessing techniques and more extensive training data to mitigate the impact of background confusion.

To improve performance, the model could benefit from enhanced preprocessing techniques, such as image segmentation, to better isolate extracellular amastigotes from the background. Additionally, increasing the number of training examples for this class would help the model learn more distinct features, further reducing misclassification. By addressing these issues, the model’s accuracy for the “extracellular amastigote” class could be improved, making it more reliable in analyzing complex cellular environments for drug discovery. As part of this effort, the dataset has been made accessible via Google Colab (https://drive.google.com/drive/folders/1X4IYxIZZ1TXysNRY1vS4zeIt30cdwuwr?usp=sharing) to encourage collaboration among researchers. Contributions from various laboratories and equipment will enhance the model’s robustness and generalizability, supporting more precise analyses, as noted by Kuznetsova et al.^15^

The F1-confidence curve (Figure 3B) is a metric that provides a detailed view of a classification model’s performance by measuring the balance between precision and recall across various confidence levels. In the overall curve (thick blue line), which represents the combined performance of all classes, it is observed that the best balance between precision and recall occurs at a maximum F1 value of 0.79 and a confidence level of 0.280. This specific confidence value plays an important role in defining thresholds that maximize the model’s overall performance, guiding the parameter settings that optimize both accuracy and sensitivity simultaneously.

The “infected cell” class (orange line) stands out with superior performance, maintaining high F1 values across a wide range of confidence levels, indicating the high effectiveness of the model in detecting this specific class. The “intracellular amastigote” class (green line) also shows robust performance, although slightly below the “infected cell” class, demonstrating itself as one of the reliable classes within the model.

The “uninfected cell” class (red line) maintains a relatively high performance in terms of F1 across several confidence levels, suggesting the model’s good capability in distinguishing this category, although slightly inferior to the “infected cell” and “intracellular amastigote” classes. In contrast, as observed earlier, the “extracellular amastigote” class (purple line) and “edge cells” class (light blue line) show more modest performance, with F1-score peaks at lower confidence levels, indicating the model’s reduced reliability in predicting these classes compared to the more critical categories. These results suggest that further refinement is necessary to enhance accuracy, particularly in distinguishing extracellular amastigotes from background noise and improving the overall model consistency across all classes.

The recall-confidence graph (Figure 3C) shows that as confidence increases, recall tends to decrease for most classes. The exception is the’infected cell’ class, which maintains a high recall level even as the confidence increases, highlighting the model’s robustness in detecting infected cells and reaffirming its central role in the system’s overall performance. On the other hand, the’intracellular amastigote’ class experiences a sharper decline in recall as confidence increases, indicating greater difficulty for the model to maintain good performance for this class at high confidence levels.

Other classes, such as “edge cells” and “extracellular amastigote,” also show a decline in recall as confidence increases. However, the “uninfected cell” class presents a more stable performance. These observations suggest adjustments in decision parameters and possibly the implementation of ensemble techniques,^16^ allowing the combination of multiple models to optimize both precision and recall simultaneously.

The precision-confidence graph (Figure 3D) shows that as confidence increases, precision improves across all classes. The “infected cell” and “uninfected cell” classes stand out, exhibiting consistently high precision, even at higher confidence levels. For the “intracellular amastigote” class, there is a significant increase in precision as confidence grows. However, it is crucial to monitor the relationship between precision and recall, as an increase in confidence may be accompanied by a sharp decline in recall, which could compromise the model’s overall performance. The “edge cells” and “extracellular amastigote” classes also show a positive trend in precision as confidence increases, albeit more gradually compared to the primary classes.

**Figure 4:**
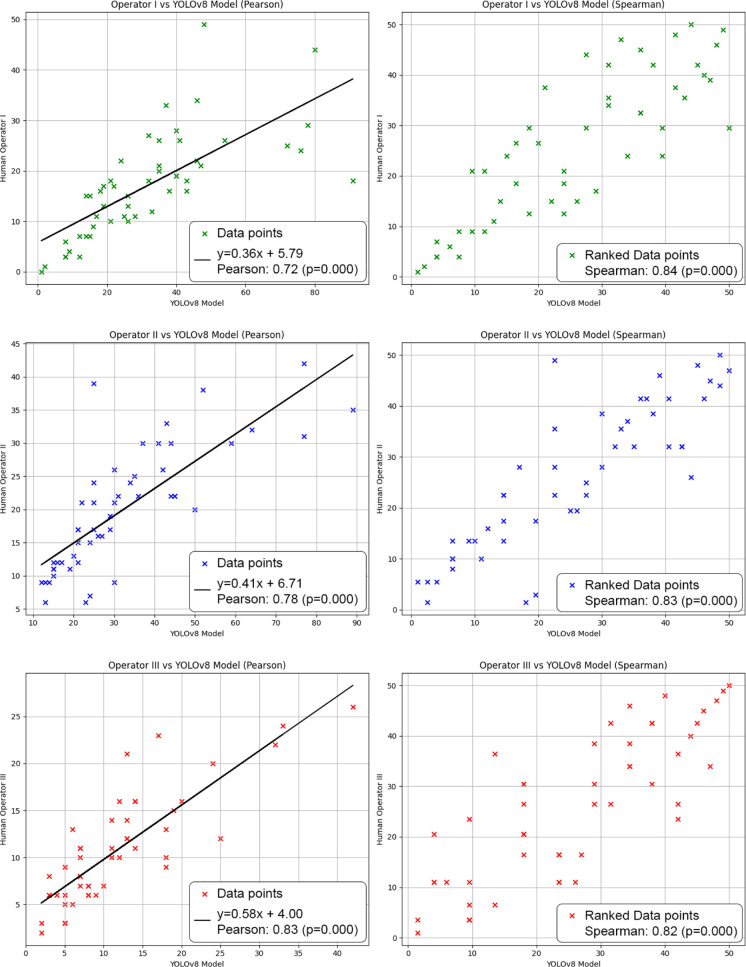
The correlation graphs between the object counts detected by three human operators and the YOLOv8 detection model. The graphs on the left illustrate the Pearson correlation, while the graphs on the right show the Spearman correlation. The Pearson correlation coefficient for operator I was 0.72 (*p* value = 4.05 × 10^–9^), and the Spearman coefficient was 0.84 (*p* value = 1.63 × 10^–14^). For operator II, the Pearson and Spearman coefficients were 0.78 (*p* value = 1.71 × 10^–11^) and 0.83 (*p* value = 5.69 × 10^–14^), respectively. For operator III, the Pearson and Spearman coefficients were 0.83 (*p* value = 1.17 × 10^–13^) and 0.82 (*p* value = 5.10 × 10^–13^). All *p* values are below 0.001.

To verify the relationship between the detection of “intracellular amastigote” by the model and human operators, Figure 4 presents the Pearson and Spearman correlation coefficients. The Pearson test was used to assess the linear correlation between the variables, while the Spearman test captured nonlinear monotonic correlations. The analysis shows that for “intracellular amastigote,” which plays a pivotal role in determining the IC_50_ curve, the model demonstrates strong alignment with human operators. This indicates that the model reliably detects the decay in amastigote counts across different drug concentrations, highlighting its utility in evaluating drug efficacy. These results reinforce the potential of the model to be integrated into preclinical assays, where accurate detection of amastigotes is crucial for determining the IC_50_ and assessing new therapeutic compounds.

The comparative graphs presented show the model’s counts represented on the *X*-axis and those of human operators on the *Y*-axis. For operator I, the Pearson correlation is 0.72, which is the lowest among the three operators, and the Spearman correlation is 0.84. The regression line slope (0.36) indicates that YOLOv8 tends to overestimate the counts compared to operator I. For operator II, the Pearson correlation is 0.78, and the Spearman correlation is 0.83, with a regression line slope of 0.41, suggesting a more pronounced overestimation than for operator I. Operator III shows the highest Pearson correlation (0.83), with a Spearman correlation of 0.82 and a slope of 0.58, indicating that while YOLOv8 still overestimates the counts, it does so to a lesser degree compared to the other operators.

The “data points” in the graphs represent the absolute counts of objects in the image, providing insight into the differences between the automated YOLOv8 estimates and manual measurements by human operators. Despite a significant correlation between the two, there is a consistent tendency of YOLOv8 to overestimate counts, as indicated by the regression line slopes. This overestimation appears to stem from the’edge cell’ class, which shows more modest performance in its confidence curve compared to other classes (Figure 3). Many training images feature numerous amastigotes, and when these amastigotes are positioned at the edges of the images, the model struggles to correctly classify them, often misclassifying them as’intracellular amastigotes.’

As seen in Figure S2, the tendencies toward overestimation reveal some discrepancies during extended image analysis, particularly after image 30 with operator I and at the beginning with operator III. However, these differences did not significantly affect the accuracy of the IC_50_ measurements, highlighting the robustness and reliability of the YOLOv8 model in minimizing human bias. The consistency in counting trends between the model and human operators suggests both respond similarly to variations in the samples, maintaining a proportional relationship. As a result, the percentage-based relationship that defines IC_50_ remains stable. Since the IC_50_ is calculated from a nonlinear regression based on the relative reduction in amastigote counts, this proportional consistency is critical for ensuring accurate IC_50_ values, even if absolute counts differ.

The “ranked data points” reflect the ordering of the counts, offering a more robust evaluation of how the YOLOv8 model captures the relative hierarchy of detected objects. Even when absolute counts vary, the model consistently preserves the ranking of detections, which maintains the linearity of the regression curve. This ensures that the IC_50_ value remains consistent, regardless of the absolute counts. This consistency is a key strength of the model, akin to requiring the same operator to evaluate different concentrations in a protocol, as each operator has a unique threshold of perception. The strong Spearman correlation between the model and human operators underscores YOLOv8’s ability to detect relative patterns effectively, even when exact counts are slightly overestimated.

Despite this strength, consistent discrepancies in counts, such as slight over- or underestimation, can be mitigated by applying correction factors or expanding the dataset with diverse human examples. By incorporating data from different operators, laboratories, and experiments, the model’s robustness can be further improved, allowing it to be standardized as a universal tool for preclinical assays. Such comprehensive training will not only reduce variability but also enhance the accuracy of IC_50_ values, ensuring that results are reliably aligned with human interpretation and critical for experimental precision.

## Conclusion

This study presents significant advancements in the application of deep learning models, particularly YOLOv8, for detecting and accurately counting Leishmania amastigotes in microscopic images. The model demonstrates robustness in identifying key categories such as “infected cells” and “intracellular amastigotes,” which are essential for evaluating drug efficacy against leishmaniasis. Despite minor overestimation issues and confusion with background elements, the model provides reliable results, aligning well with human evaluations. By offering real-time detection and the potential for collaborative dataset expansion through the Google Colab platform, this approach enhances the speed and accuracy of preclinical assays. Future work will focus on refining model performance by addressing the challenges posed by smaller classes such as “extracellular amastigotes” and background noise, ensuring broader applicability in drug discovery processes.

## Methods

### Categories for Evaluating Antileishmanial Activity

The study relied on the accurate quantification of the “infected cell” and “intracellular amastigote” categories to assess the antileishmanial activity of new compounds. The “infected cell” category was used to indicate the extent of infection by identifying the presence of Leishmania within host cells, while the “intracellular amastigote” category measured the parasitic load itself. Additional categories – uninfected cells, extracellular amastigotes, and edge cells – were included to improve classification accuracy. “Uninfected cells” distinguish healthy cells from infected ones, “extracellular amastigotes” identify parasites outside the host cell, and “edge cells” reduce errors from partial cell visualization. The “Background” category, though not explicitly labeled or learned directly, plays a critical role by representing regions of the image that do not belong to any of these predefined classes. It ensures that areas irrelevant to the detection process are accurately excluded, thus minimizing confusion and ensuring precise classification. These categories together help ensure robust analysis by reducing misclassification and improving the overall accuracy of the detection model.

### Analytical Slides for Deep Learning Training and Human Validation

The preparation of analytical slides, as outlined in the referenced study,^17^ plays a key role in training deep learning models and human validation for amastigote detection and quantification. Briefly, this process involves treating Leishmania-infected cells with varying concentrations of test compounds, dehydrating the coverslips with acetone and xylene, and staining them with Giemsa (1:10 in distilled water) to enhance visibility. These slides are analyzed under optical microscopy, with 200 cells per coverslip counted to ensure statistical reliability. The manually counted results provide the training dataset for the deep learning model, while also serving as a validation reference to ensure consistency with expert evaluations.

### Microscopic Image Acquisition and Processing for Model Training

For this analysis, the YOLOv8 model was trained with 470 images, each containing distinct examples of Leishmania amastigote cells, necessary for the neural network’s learning process. These images include the detection of different morphologies of Leishmania amastigote cells, categorized into the following classes: infected cells, intracellular amastigotes, uninfected cells, extracellular amastigotes, and edge cells. These classes were defined for application in microscopy based on real patterns observed during routine monitoring conducted by the research group in evaluating the efficacy of new drugs against leishmaniasis.

The images were captured using Leica DM5500 B and Nikon Eclipse Ci microscopes, along with Leica DFC495 and Moticam Pro cameras. After image capture, each class of interest was annotated using the LabelMe software, which allows for object marking in images and creates the annotated dataset for deep learning model training.^18^

**Figure 5:**
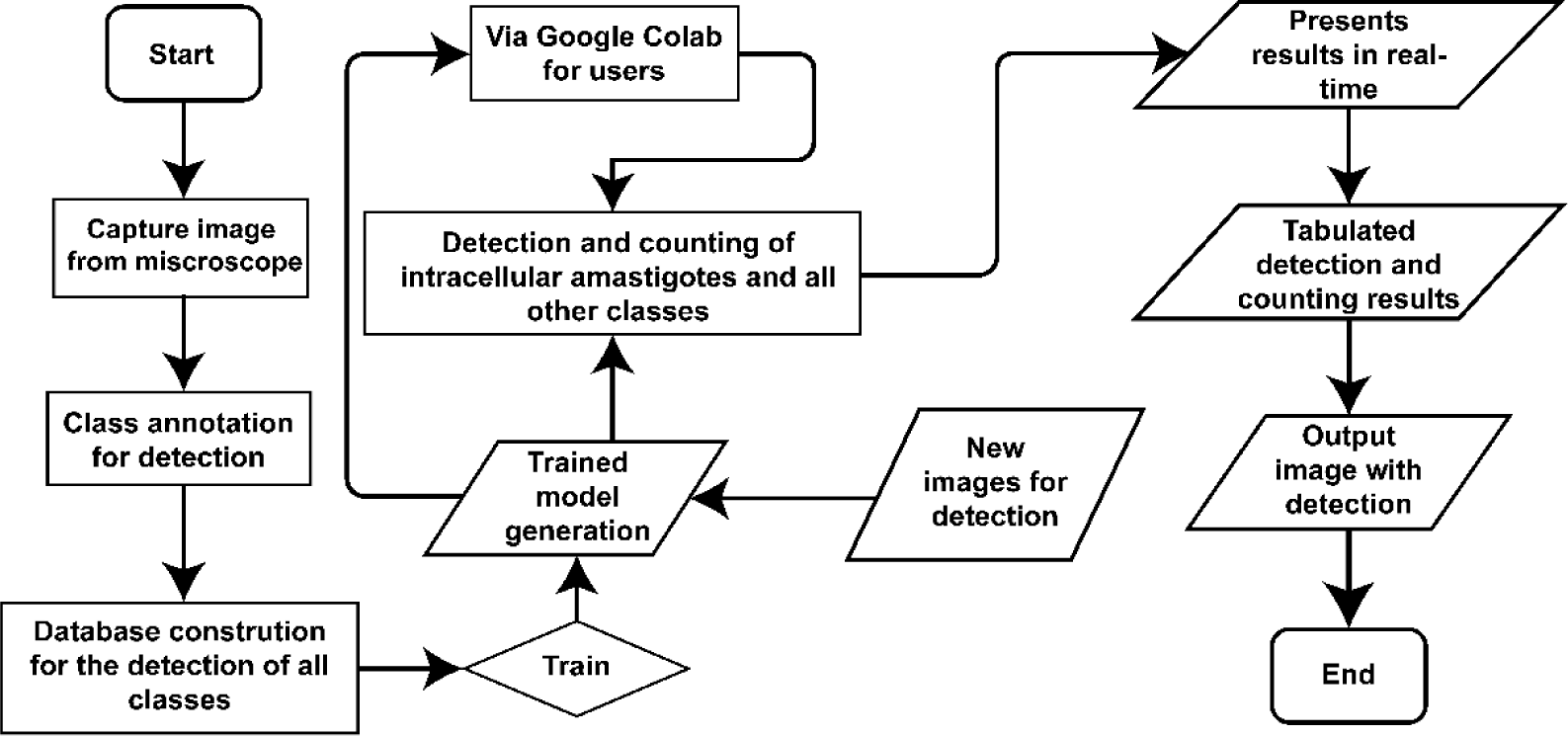
Flowchart illustrates the process from microscopic image capture and annotation of the classes of interest to database construction, YOLOv8 model training, and detection of new images. After training, the model was made available via Google Colab, allowing users to perform automatic detection and counting of their images. The results are presented in real-time using a USB camera in the form of tables or images containing the detections.

The “overview of the automatic tracking algorithm process” illustrates the entire workflow from image acquisition to data interpretation by the trained model, including automatic detection performed by YOLOv8. Figure 5

The YOLOv8 model was trained for 100 epochs using loss patterns to evaluate the learning curve and identify the saturation point in the optimization process. During training, box loss, classification loss (cls loss), and distance from boundary regression loss (dfl loss) metrics were monitored to analyze the behavior of the loss function. Over the epochs, the total loss approached to one. According to Bengio,^19^ as training progresses, the model may reach a point where optimization slows and improvements in the loss function become progressively smaller. To improve generalization and avoid overfitting, excessive adjustment of the model to the training data, various data augmentation techniques were applied, artificially expanding the training dataset.^20^ The techniques used included random scale, erasing regions, adding noise, mosaic, random contrast, vertical and horizontal flip, tile generation, crop resizing, and gamma adjustment.

### Model Performance Evaluation

Precision-recall, F1-confidence, Recall-confidence, and Precision-confidence curves were employed. The precision-recall curve is particularly useful in class-imbalanced scenarios, such as rare object detection, by illustrating the balance between precision (the proportion of correct predictions among all predictions) and recall (the percentage of correct detections among all actual instances).^21^ The F1-confidence metric combines precision and recall into a single value, providing a balanced assessment of the model’s performance. The recall-confidence and precision-confidence curves, on the other hand, allow for the observation of how model performance shifts with varying confidence thresholds, helping in fine-tuning the optimal cutoff point for decision-making in practical applications.^22^

Recall measures the model’s capacity to detect all true positive cases (i.e., intracellular amastigotes), making it crucial in applications like pathogen detection, where minimizing false negatives is essential. Precision ensures that the predictions made by the model are mostly accurate by calculating the proportion of true positives among all predicted positives. The F1-score balances both recall and precision, particularly useful in data sets with imbalanced classes, such as distinguishing infected from noninfected cells. Confidence, meanwhile, refers to the model’s assigned probability to a given prediction, indicating the certainty behind each prediction. Together, these metrics provide comprehensive insight into the model’s performance across different thresholds and conditions.

### Comparison between Human Direct Counts and YOLOv8 Model Predictions

Subsequently, with the trained model, three qualified operators conducted collections on different days, aiming to analyze and count “intracellular amastigotes.” Each operator examined 50 images, corresponding to 50 microscopic fields, and performed the counts directly through the microscope rather than on the images captured by the camera. This procedure follows the standard in microscopic analyses,^17^ as direct observation through the microscope, leveraging human perception, allows for a clearer and more detailed view of cellular structures, where certain details may be easier to discern. During the counting process of each microscopic field, a photo of that field was taken, and the YOLOv8 model made predictions based on the image. These images, not used during training, served as a validation set to test the model’s performance on previously unseen data.^23^ The counts conducted by the operators were then quantitatively compared to the results obtained with the neural network.

### Real-Time Detection Test with the HY-500 M Camera

To test the real-time detection capability of the YOLOv8 model on microscopic images, the HY-500 M 5MP USB digital camera was used, attached to the eyepiece tube of a compatible biological microscope. The HY-500 M camera was selected for its ability to capture images directly on a computer. It was installed on the eyepiece tube using compatible adapters for different diameters, such as 23.2 mm, 30 mm, and 30.5 mm. The camera cost approximately 25 dollars.

The camera was connected to the computer via USB, and the analysis process was performed directly in Google Colab, where the YOLOv8 model was already running. As soon as the image was captured by the camera, the model automatically detected *Leishmania* amastigotes in real time, even before the photo was saved. During the process, lighting and contrast conditions were adjusted directly on the microscope to optimize the capture of relevant morphological details for automatic detection.

### Statistical Evaluation of Cross-Validation

For cross-validation of the YOLOv8 model, 20% of the dataset was used, with samples stratified to ensure representation of all classes in the validation set. The cross-validation technique is widely recognized for providing an accurate assessment of model performance, as it helps detect overfitting and provides a more realistic estimate of how the model will perform on unseen data.^24^

Additionally, a confusion matrix was used to calculate true positives (TP), true negatives (TN), false positives (FP), and false negatives (FN). The choice of the confusion matrix allows for the evaluation of model performance in terms of classification hits and errors for each class, providing insights for potential adjustments.^25^

To evaluate the model’s performance relative to human operators, Pearson and Spearman correlation analyses were conducted, considering both the detection and counting of “intracellular amastigotes” in the images. Additionally, scatterplots were generated to visualize the absolute and relative counts between the model and the operators, allowing for a graphical analysis of the relationship between the variables. Statistical significance indices were also calculated to verify whether the observed correlations were statistically significant.

## Supporting Information Available

Connection setup of the microscope and laptop via USB cable. The laptop shows the Google Colab interface with real-time detection of intracellular amastigotes and other classes using the YOLOv8 image analysis model, Comparative graphs of Leishmania amastigote counts by the YOLOv8 model and three human operators (I, II, and III) for 50 images. The YOLOv8 counts are represented by blue lines, and the human operator counts by orange lines, illustrating variations across the images (PDF)

## References

[ref1] aWHO. World Health Organization - Leishmaniasis. 2023. https://www.who.int/news-room/fact-sheets/detail/leishmaniasis (Accessed 09 June 2024).

[ref2] De Rycker M.; Wyllie S.; Horn D.; Read K. D.; Gilbert I. H. Anti-trypanosomatid drug discovery: Progress and challenges. Nat. Rev. Microbiol. 2023, 21 (1), 35–50. 10.1038/s41579-022-00777-y. 35995950 PMC9395782

[ref3] aMann S.; Frasca K.; Scherrer S.; Henao-Martinez A. F.; Newman S.; Ramanan P.; Suarez J. A. A Review of Leishmaniasis: Current Knowledge and Future Directions. Curr. Trop. Med. Rep. 2021, 8 (2), 121–132. 10.1007/s40475-021-00232-7. 33747716 PMC7966913

[ref4] Bruschi F.; Gradoni L. The leishmaniases: Old neglected tropical diseases; Springer, 2018.

[ref5] aCallahan H. L.; Portal A. C.; Devereaux R.; Grogl M. An axenic amastigote system for drug screening. Antimicrob. Agents Chemother. 1997, 41 (4), 818–822. 10.1128/AAC.41.4.818. 9087496 PMC163801

[ref6] Dias-Lopes G.; Zabala-Peñafiel A.; de Albuquerque-Melo B. C.; Souza-Silva F.; Menaguali Do Canto L.; Cysne-Finkelstein L.; Alves C. R. Axenic amastigotes of Leishmania species as a suitable model for in vitro studies. Acta Trop. 2021, 220, 105956 10.1016/j.actatropica.2021.105956. 33979642

[ref7] Salem M. M.; Werbovetz K. A. Natural products from plants as drug candidates and lead compounds against leishmaniasis and trypanosomiasis. Curr. Med. Chem. 2006, 13 (21), 2571–2598. 10.2174/092986706778201611. 17017912

[ref8] Zulfigar B.; Shelper T. B.; Avery V. M. Leishmaniasis drug discovery: Recent progress and challenges in assay development. Drug Discovery Today 2017, 22 (10), 1516–1531. 10.1016/j.drudis.2017.06.004. 28647378

[ref9] Sadeghi A.; Sadeghi M.; Fakhar M.; Zakariaei Z.; Sadeghi M.; Bastani R. A deep learning-based model for detecting Leishmania amastigotes in microscopic slides: A new approach to telemedicine. BMC Infect. Dis. 2024, 24 (1), 551 10.1186/s12879-024-09428-4. 38824500 PMC11144338

[ref10] Tekle E.; Dese K.; Girma S.; Adissu W.; Krishnamoorthy J.; Kwa T. DeepLeish: A deep learning based support system for the detection of Leishmaniasis parasite from Giemsa-stained microscope images. BMC Med Imaging 2024, 24 (1), 152 10.1186/s12880-024-01333-1. 38890604 PMC11186139

[ref11] aSrivastava S.; Divekar A. V.; Anilkumar C.; Naik I.; Kulkarni V.; Pattabiraman V. Comparative analysis of deep learning image detection algorithms. J. Big Data 2021, 8 (1), 66 10.1186/s40537-021-00434-w.

[ref12] Aljohani A. Predictive Analytics and Machine Learning for Real-Time Supply Chain Risk Mitigation and Agility. Sustainability 2023, 15, 15088 10.3390/su152015088.

[ref13] Gonçalves C.; Borges A.; Dias V.; Marques J.; Aguiar B.; Costa C.; Silva R. Detection of human visceral leishmaniasis parasites in microscopy images from bone marrow parasitological examination. Appl. Sci. 2023, 13 (14), 8076 10.3390/app13148076.

[ref14] Sun C.; Xu A.; Liu D.; Xiong Z.; Zhao F.; Ding W. Deep Learning-Based Classification of Liver Cancer Histopathology Images Using Only Global Labels. IEEE J. Biomed. Health Inf. 2020, 24 (6), 1643–1651. 10.1109/JBHI.2019.2949837. 31670686

[ref15] Kuznetsova A.; Rom H.; Alldrin N.; Uijlings J.; Krasin I.; Pont-Tuset J.; Kamali S.; Popov S.; Malloci M.; Kolesnikov A.; et al. The Open Images Dataset V4. Int. J. Comput. Vis. 2020, 128 (7), 1956–1981. 10.1007/s11263-020-01316-z.

[ref16] Dietterich T. G. Ensemble methods in machine learning. In International workshop on multiple classifier systems, 2000; Springer, pp 1–15, 2014.

[ref17] da Silva F.; Rizk Y. S.; Das Neves A. R.; Lourenço E. M. G.; Ferreira A. M. T.; Monteiro M. M.; de Lima D. P.; Perdomo R. T.; Bonfá I. S.; Toffoli-Kadri M. C.; et al. Antileishmanial Activity, Toxicity and Mechanism of Action of Complexes of Sodium Usnate with Lanthanide Ions: Eu(III), Sm(III), Gd(III), Nd(III), La(III) and Tb(III). Int. J. Mol. Sci. 2024, 25 (1), 413 10.3390/ijms25010413. PMC10779311 38203584

[ref18] Russell B. C.; Torralba A.; Murphy K. P.; Freeman W. T. LabelMe: A database and web-based tool for image annotation. Int. J. Comput. Vis. 2008, 77, 157–173. 10.1007/s11263-007-0090-8.

[ref19] Bengio Y. Practical Recommendations for Gradient-Based Training of Deep Architectures. In Neural networks: Tricks of the trade, Second ed.; Springer, 2012; pp. 437–478.

[ref20] Shorten C.; Khoshgoftaar T. M. A survey on image data augmentation for deep learning. J. Big Data 2019, 6 (1), 60 10.1186/s40537-019-0197-0. PMC8287113 34306963

[ref21] Davis J.; Goadrich M. The relationship between Precision-Recall and ROC curves. In Proceedings of the 23rd international conference on Machine learning,ACM 2006.

[ref22] Saito T.; Rehmsmeier M. The precision-recall plot is more informative than the ROC plot when evaluating binary classifiers on imbalanced datasets. PLoS One 2015, 10 (3), e0118432 10.1371/journal.pone.0118432. 25738806 PMC4349800

[ref23] Heaton J. Ian Goodfellow, Yoshua Bengio, and Aaron Courville: Deep learning. Genet. Program. Evolvable Mach. 2018, 19 (1), 305–307. 10.1007/s10710-017-9314-z.

[ref24] Kohavi R. A study of cross-validation and bootstrap for accuracy estimation and model selection. In Proceedings of the 14th international joint conference on Artificial intelligence Montreal, IJCAI 1995.

[ref25] Sokolova M.; Lapalme G. A systematic analysis of performance measures for classification tasks. Inf. Process. Manag. 2009, 45 (4), 427–437. 10.1016/j.ipm.2009.03.002.

