# Supplementary material for: Human-Validated Neural Networks for Precise Amastigote Categorization and Quantification to Accelerate Drug Discovery in Leishmaniasis
Source: ACS Omega. 2024 Dec 24;10(1):1177–87. doi: 10.1021/acsomega.4c08735 (PMC11740139; doi:10.1021/acsomega.4c08735)
Supplement: Supplementary file 1 — ao4c08735_si_001.pdf [file ao4c08735_si_001.pdf]

Human-Validated Neural Networks for Precision Amastigote Categorization and Quantification to Accelerate Drug Discovery in Leishmaniasis

Andrey Gaspar Sorrilha-Rodrigues[1], João Lucas Aparecido Rocha Paes[2], Yasmin Silva Rizk[3], Fernanda da Silva[3], Rafael Francisco Rosalem[3], Carla Cardozo Pinto de Arruda[3], Carlos Alexandre Carollo[1]

1 - Laboratory of Natural Products and Mass Spectrometry (LAPNEM), Faculty of Pharmaceutical Sciences, Food, and Nutrition (FACFAN), Federal University of Mato Grosso do Sul, Campo Grande, Mato Grosso do Sul, Brazil; 2 - Geomatics Laboratory - georeferencing and computer vision, Faculty of Computing, Federal University of Mato Grosso do Sul, Campo Grande, Mato Grosso do Sul, Brazil; 3 - Human Parasitology Laboratory, Institute of Biosciences, Federal University of Mato Grosso do Sul, Campo Grande, Mato Grosso do Sul, Brazil.

Corresponding Author: carlos.carollo@ufms.br

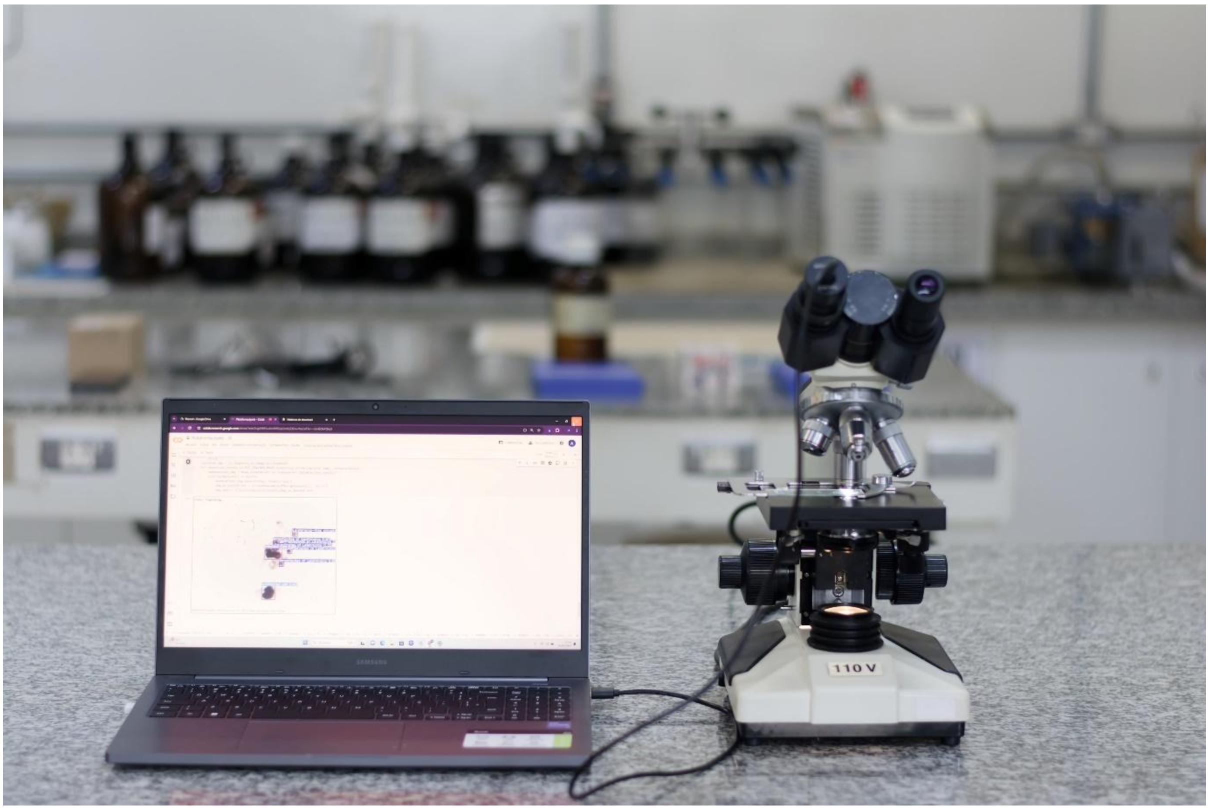

Figure S1. The microscope is connected to the laptop via a USB cable, with the laptop displaying the Google Colab interface. The system performs real-time detection of intracellular amastigotes and other cell categories using the YOLOv8 image analysis model. The captured images are processed directly from the microscope, enabling on-the-fly detection and classification of objects.

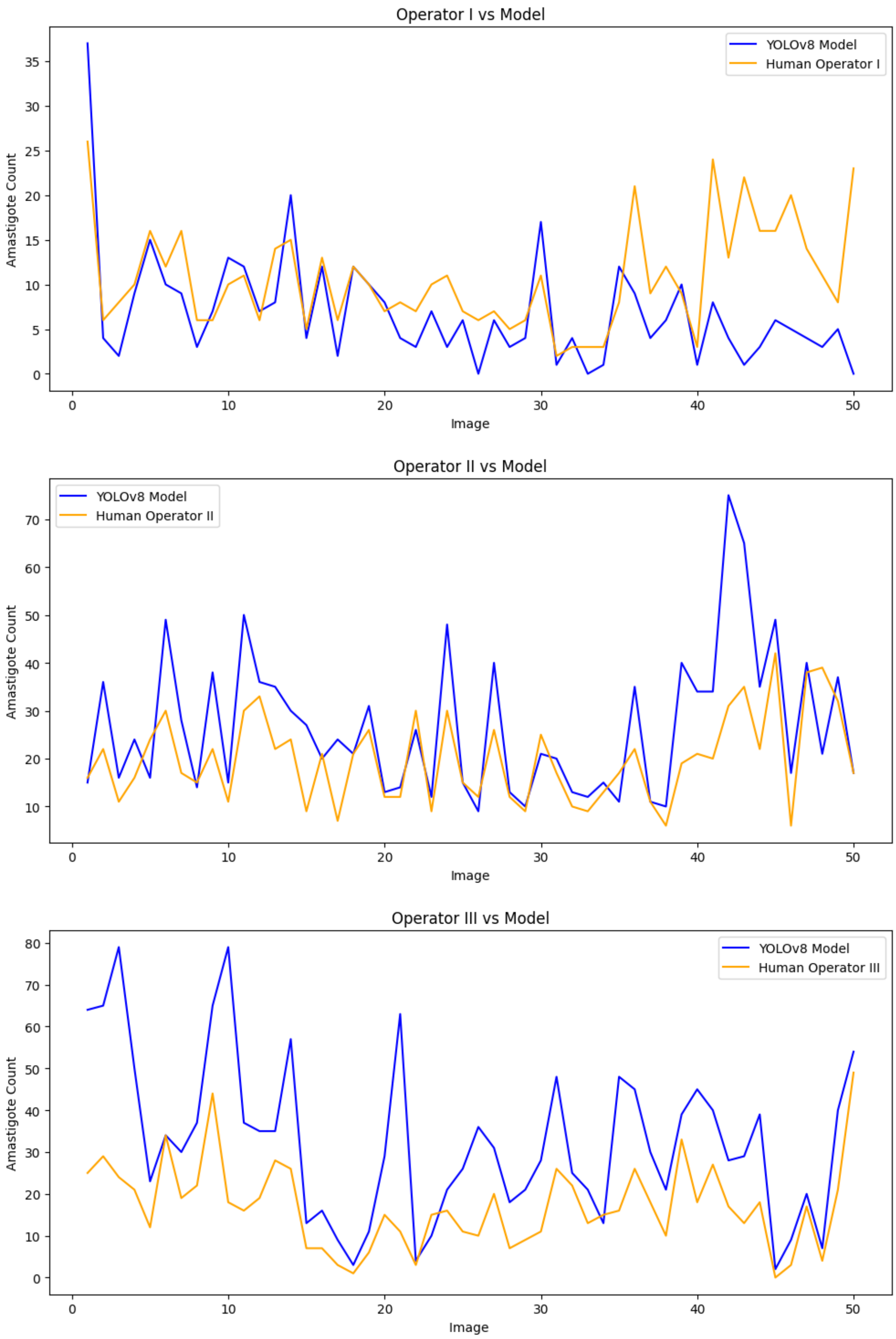

Figure S2. The graphs compare the *Leishmania* amastigote counts performed by the YOLOv8 model and three human operators (I, II, and III) for 50 images. The blue lines represent the counts performed by YOLOv8, while the orange lines represent the counts by the human operators. Variations in the counts are displayed across the 50 images.
